# Electroencephalographic studies in growth-restricted and small-for-gestational-age neonates

**DOI:** 10.1038/s41390-022-01992-2

**Published:** 2022-02-23

**Authors:** Nathan J. Stevenson, Melissa M. Lai, Hava E. Starkman, Paul B. Colditz, Julie A. Wixey

**Affiliations:** 1 Brain Modelling Group, QIMR Berghofer Medical Research Institute, Brisbane, QLD Australia; 2 UQ Centre for Clinical Research, Faculty of Medicine, The University of Queensland, Herston, QLD 4029 Australia; 3 Perinatal Research Centre, Royal Brisbane and Women’s Hospital, Herston, QLD 4029 Australia; 4 Department of Obstetrics and Gynaecology, University of Toronto, King’s College Circle, Toronto, ON M5S Canada

## Abstract

**Abstract:**

Foetal growth restriction (FGR) and being born small for gestational age (SGA) are associated with neurodevelopmental delay. Early diagnosis of neurological damage is difficult in FGR and SGA neonates. Electroencephalography (EEG) has the potential as a tool for the assessment of brain development in FGR/SGA neonates. In this review, we analyse the evidence base on the use of EEG for the assessment of neonates with FGR or SGA. We found consistent findings that FGR/SGA is associated with measurable changes in the EEG that present immediately after birth and persist into childhood. Early manifestations of FGR/SGA in the EEG include changes in spectral power, symmetry/synchrony, sleep–wake cycling, and the continuity of EEG amplitude. Later manifestations of FGR/SGA into infancy and early childhood include changes in spectral power, sleep architecture, and EEG amplitude. FGR/SGA infants had poorer neurodevelopmental outcomes than appropriate for gestational age controls. The EEG has the potential to identify FGR/SGA infants and assess the functional correlates of neurological damage.

**Impact:**

- FGR/SGA neonates have significantly different EEG activity compared to AGA neonates.
- EEG differences persist into childhood and are associated with adverse neurodevelopmental outcomes.
- EEG has the potential for early identification of brain impairment in FGR/SGA neonates.

## Introduction

Foetal growth restriction (FGR) is a failure of the foetus to meet normative in utero growth potential. It is defined using umbilical artery doppler, birth weight for age, physiological determinants, neonatal features of malnutrition, and in utero growth retardation.^1,2^ It differs from a definition of small for gestational age (SGA) which is typically defined as a birth weight less than the 10th percentile for a given gestational age, irrespective of any pathology. Nevertheless, being born either FGR or SGA is associated with neurodevelopmental delay.^3,4^

FGR is commonly caused by placental insufficiency, resulting in inadequate delivery of oxygen and nutrients from the placenta to the developing foetus. FGR newborns are at an increased risk of mortality and morbidity.^5^ Chronic nutritional deficiencies have been shown to impair brain development in FGR infants^6–9^ with adverse long-term neurological outcomes including language delays, learning, and behavioural problems, and cerebral palsy (CP).^3,10–12^ FGR infants are 5–30 times more likely to develop CP.^10,13^ FGR infants born <35 weeks gestation score lower than appropriate for gestational age (AGA) children across a range of neurodevelopmental assessments^14^ with neurodevelopmental delays reported in 24–53% of FGR infants at 2 years of age.^15,16^

Many babies with FGR are undiagnosed until the time of birth.^17^ Neuroimaging studies, predominantly ultrasound and magnetic resonance imaging (MRI), have been investigated as potential methods of screening for neonatal brain injury. Cranial ultrasounds are readily available, however, rely on operator proficiency and lack sensitivity when detecting subtle or diffuse brain injuries.^18^ Neonatal MRIs are more sensitive and can detect subtle neuropathology by high-resolution visualisation of structural changes.^19^ Imaging studies have shown persistent structural brain deficits in infants with FGR that remain at 1 year of age.^19,20^ However, implementing MRI screening on a large scale is costly and resource intensive.

Electroencephalography (EEG) is a useful clinical tool for the early identification of adverse brain outcomes in the neonatal population.^21–23^ EEG is a well-established method that is non-invasive and monitors changes in real time. It can continually measure cortical function over long periods of time and is commonly used as an aid in the assessment of seizures, sleep, and functional neurological outcomes.^24,25^ It also reveals clear, distinct changes with brain maturation in infants, children, and adolescents^26,27^ and has been demonstrated to be a predictor of later neurodevelopmental deficits in preterm infants.^28^ However, its routine use towards optimising neurodevelopment outcomes in this cohort is impeded by the tendency for artefact affected recordings and limited resources in its clinical interpretation.^29^ In this review, we summarise the literature on the effects of FGR/SGA on the EEG. We collate findings on EEG and amplitude-integrated EEG (aEEG) changes in FGR/SGA infants over a range of gestational ages (GA) from immediately after birth into early childhood.

## Methods

### Search category—EEG studies on FGR/SGA neonates

A review of the literature was performed using PubMed, BMJ, Cochrane Library, CINAHL, Embase, and Web of Science. Inclusion criteria: (1) published in a peer-reviewed journal on the specific topic of EEG on FGR/SGA neonates with both FGR/SGA and control (AGA) groups included in the study and (2) published in English. There was a total of 35 studies, and 7 met the inclusion criteria. An additional paper was found by reviewing the reference lists from the papers included. Assessment of study quality was undertaken using the Strengthening the Reporting of Observational Studies in Epidemiology statement checklist STROBE.^30^ Each checklist item was categorised as ‘yes’ (met the criteria), ‘no’ (did not meet the criteria), or ‘not applicable’. Each manuscript was reviewed by two co-authors (JW and NS). Each reviewer evaluated the article content independently. Discrepancies were resolved by consensus among the reviewers. Due to the limited number of studies and heterogeneity of outcome measures in the studies, meta-analysis was not undertaken.

### Early aEEG studies on FGR/SGA neonates

The aEEG is a time and amplitude compressed summary measure, or trend, of the EEG. It is typically recorded with a limited number of electrodes (2 or 4 electrodes placed, most commonly, at frontal, central or parietal regions). It is simple, easy to interpret, and commonly used for brain monitoring in the neonatal intensive care unit (NICU).^31^ The aEEG is typically interpreted using visual inspection of the lower and upper margins of the aEEG trend over a period of 3–6 h at a time. The values of these margins can be evaluated directly or used to define distinct patterns such as continuous normal voltage, discontinuous, burst suppression, and seizure. The evolution of the aEEG over time can also be used to assess the presence or absence of sleep–wake cycling (SWC; the oscillation between periods of awake, indeterminate, quiet, and active sleep). These measurements can further be combined into a single representative score that is used for prognostication.^32,33^ There is evidence that supports improved long-term outcomes with the presence of SWC within the first few days of life in preterm infants.^28^ We found four studies that used aEEG to assess the effect of FGR/SGA on cortical function (Table 1). These studies encompass extremely preterm (<28 weeks), very preterm (28–32 weeks), and moderate to late preterm (32–37 weeks) FGR/SGA and AGA neonates.^34^ As FGR infants are a subgroup of SGA infants, we report and analyse separately with similarities drawn where appropriate.

**Table 1:** Summary of aEEG studies of FGR/SGA neonates.

Study	aEEG channel	Population (*N*)	Mean GA	PMA of EEG	SWC	aEEG grade
Schwindt et al., 2015	One channel (P3–P4)	p-SGA (47)	27.5 weeks*	<14 days of life	**↓**65% present*	C/D/BS11%/40%/21%
		p-AGA (89)	26 weeks		96% present	C/D/BS8%/53%/25%
Benavente-Fernandez et al., 2017	Two biparietal channels (C3–P3, C4–P4)	p-SGA (18)	28.2 ± 2.7 weeks	12–14 h of life; 46–48 h; 70–72 h	**↑**50% present***	DHV/C**↑**80%***/**↑**71%**/**↑**83%*12 h/24–48 h/72 h
		p-AGA (74)	27.9 ± 2.2 weeks		15% present	DHV/C50%/51%/70%2 h/24–48 h/72 h
Griesmaier et al., 2015	One channel (P3–P4)	p-SGA (50)	29.57 weeks (27.89; 30.71)	18–24 h; 42–48 h; 66–72 h; day 7; day 14, day 21, day 28		C37%/50%/55%/88%d1/d3/w1/w4
		p-AGA (255)	30 weeks (28.14; 31.14)			C34%/52%/57%/88%d1/d3/w1/w4
Yerushalmy-Feler et al., 2014	One channel (C3–C4)	p-FGR (14)	34.3 ± 1.8 weeks	Within first 48 h life	50% present	C**↓**74%**
		p-AGA (16)	33.7 ± 2 weeks		31% present	C92%

**↑** increased, **↓** decreased in FGR/SGA in comparison to AGA.

*GA* gestational age, *PMA* postmenstrual age, *SWC* sleep–wake cycle, *C* continuous, *D* discontinuous, BS burst suppression, prefix *t* term, prefix *p* preterm.

*p < 0.05; **p < 0.01; ***p < 0.001.

Benavente-Fernadez et al. showed that immediately after birth (within 12 h), a significantly higher percentage of extremely and very preterm SGA neonates with normal neurodevelopment had developed SWC compared to AGA controls (SGA 50%; AGA 15%).^9^ The selection of SGA infants with a good prognosis attempts to define a ‘brain spared’ group. In growth-restricted infants, brain sparing is a foetal response to limited resources and involves a redistribution of blood flow to vital organs (such as the brain) at the expense of other body parts resulting in asymmetric growth restriction. Although brain sparing is considered a protective mechanism in FGR, recent evidence has shown asymmetric FGR infants may have worse neurodevelopmental outcomes compared with symmetric FGR infants.^8,14,35–39^ Furthermore, not all SGA infants are growth restricted and may be developing along a lower, but normal, growth trajectory.^2^ This study raises the interesting prospect of EEG monitoring immediately after birth as a form of evoked potential where the analysis time is hours rather than seconds. Many aEEG/EEG studies focus on the immediate postnatal period in the search for diagnostic and prognostic markers.

Schwindt et al. showed that within a week, in a cohort with similar GA, SWC is more apparent on the aEEG, in general, but the trend reverses with SWC more prevalent in AGA controls (SGA 65%; AGA 96%). A trend that alters but holds when infants without sedatives and cerebral lesions are removed (SGA 84%; AGA 97%).^33^

Griesmaier et al. showed no differences in aEEG continuity between SGA and AGA very preterm neonates when recorded within the first 72 h of life;^40^ although SGA neonates were found to have a significantly increased number of aEEG bursts per hour within 24 h of birth (SGA 17.4/h; AGA 10.1/h). The percentage of time spent in a combined, continuous/discontinuous high voltage pattern was increased in preterm SGA neonates with these differences being apparent in the immediate post-natal period (within 72 h of life).^9^ However, no differences in aEEG background patterns were reported in extremely preterm SGA neonates by the second week of life; although SGA neonates were more likely to have seizures than AGA controls.^33^

In a moderate to late preterm FGR cohort, Yerushalmy-Feler et al. examined SWC and reported a similar, but not significant, trend towards a high proportion of established SWC in FGR neonates (FGR 50%; AGA 31%) in the aEEG recorded within 48 h of birth. Yerulshamy-Feler et al. also showed a significant decrease in aEEG continuity (FGR 74% ± 17; AGA 92% ± 16) in moderate to late preterm FGR neonates within 48 h of life compared with AGA controls.^41^

The findings of aEEG studies suggest that during a period of post-natal adaption an increased proportion of SGA infants (<32 weeks GA) establish normal aEEG activities such as SWC and continuity more rapidly than AGA controls, resulting in more apparent maturity. These effects of post-natal adaption are, however, reduced as GA increases. Abnormal aEEG activity (lack of SWC) appear in SGA/FGR infants as post-natal age increases.^33^

### EEG studies on FGR/SGA neonates

The EEG is recorded using either a full 10–20 international system (19–21 electrodes) or a modified version for neonates (9–12 electrodes). In addition to the characteristics observed in the aEEG, the EEG is visually interpreted for patterns associated with normal maturation, such as trace discontinue, temporal theta, trace alternant, synchrony/symmetry, and inter-burst interval, or abnormal function, such as mechanical delta brushes, spikes/sharp waves, and seizures.^42^ We found three studies that use EEG to assess the effect of FGR/SGA on cortical function (Table 2).

**Table 2:** Summary of EEG studies of FGR/SGA neonates.

Study	EEG channel	Popn (*N*)	Mean GA	PMA of EEG	SWC	Relative delta power	Bursts	ASYM and ASYNC	Amplitude
Castro Conde et al., 2020	10–20 system	t-SGA (50)	37.7 ± 1.7 weeks	48–72 h of life	**↓**TA: 26%**, **↑**D: 38%**	↓~48%**	**↑**DB: 24%**, **↑**IBIm = 7 s**, **↑**T/h = 18**	**↑**ASYM 12%**, **↑**ASYNC 11%**	
		t-AGA (44)	38.1 ± 2.0 weeks		TA: 52%, D: 6%	~50%	DB: 6%, IBIm: 3 s, T/h: 8	ASYM 3%, ASYNC 5%	
Ozdemir et al., 2009	10–20 system	t-SGA (40)	39.4 ± 0.8	First week of life		**↓**84%* at CzC4			**↓**27.7***
		t-AGA (20)	39.7 ± 0.7			88%			63.4
Yerushalmy-Feler et al., 2014	(C3–C4)	p-FGR (14)	34.3 ± 1.8 weeks	Within first 48 h life	50% present	**↑**86% ± 3**			
		p-AGA (16)	33.7 ± 2 weeks		31% present	79% ± 5			

**↑** increased, **↓** decreased in FGR/SGA in comparison to AGA.

*GA* gestational age, *PMA* postmenstrual age, *SWC* sleep–wake cycle, *TA* trace alternant, *D* discontinuity, *DB* bursts with delta brushes, *IBIm* maximum interburst interval, *T/h* transients/bursts per hour, *ASYM* asymmetry, *ASYNC* asynchrony, prefix *t* term, prefix *p* preterm.

*p < 0.05; **p < 0.01; ***p < 0.001.

In term neonates recorded within 72 h of life, Castro Conde et al. found spectral differences in periods of the EEG with alternating or discontinuous patterns (patterns predominantly associated with indeterminate or quiet sleep) between SGA and AGA controls.^43^ SGA neonates had a lower relative delta power (averaged across all EEG channels) and subsequent increase in alpha and beta powers compared to AGA neonates. Ozdemir et al. found similar significant decreases in relative delta power and increases in relative alpha and beta powers in quiet sleep of term SGA neonates recorded within the first week of life; these changes were only significant in central channels (Cz-C4). Ozdemir et al. also found large decreases in EEG amplitude (averaged across all EEG channels) within rapid eye movement (REM) sleep in SGA neonates compared to AGA controls.^44^ Yerushalmy-Feler et al. showed increased relative delta power, and, subsequently, decreased relative theta, alpha, and beta power, in preterm FGR neonates compared to AGA controls.

Castro Conde et al. found differences in several other aspects of the EEG in SGA neonates compared to AGA controls. The ratio of discontinuous to trace alternant activity, percentage of asynchronous and asymmetric EEG activity, the percentage of EEG activity containing delta brushes, maximum inter-burst interval, and the number of transients per hour was all higher in SGA neonates.^43^

The results of spectral analyses are not always consistent across studies. Nevertheless, significant differences in EEG spectra were found between SGA/FGR and AGA infants. Other key phenomenological characteristics of the EEG such as inter-burst interval, asymmetry/asynchrony, and delta brushes were higher, and by association, underdeveloped or immature in term SGA neonates compared to AGA controls.

Phenomenological aEEG/EEG patterns are typically interpreted with respect to age (as either mature or immature). This interpretation can be complicated as several EEG patterns are abnormal, independent of age, such as seizure, positive Rolandic sharp waves, or isoelectric activity. While interpretations with respect to age are useful, key maturational EEG characteristics when quantified, explain considerably <50% of the variation in age.^27^ The remaining 50% are explained by a range of factors including EEG recording methods, biological variability, various neonatal exposures, vigilance/sleep state as well as underlying changes in brain development. It is the last factor that is of interest but is often difficult to tease out.

Future research must focus on improving the evidence base on the relationship between EEG changes and brain function. Improved understanding of this relationship through the integration of EEG findings and findings from other modalities such as MRI (infants with FGR have altered grey matter volumes, myelination, cortical complexity, hippocampal and cerebellar development, fractional anisotropy, and connectivity^45^) can improve our understanding of how growth restriction affects the developing brain.

### EEG studies of FGR/SGA neonates during infancy and childhood

The EEG has also been used to assess the neurodevelopment of FGR/SGA neonates at later stages of life. We found three studies that used EEG to assess the effect of FGR/SGA on cortical function during infancy and childhood (Table 3). These studies have shown that alterations in cortical activity in FGR/SGA neonates recorded by the EEG persist into early childhood.

**Table 3:** Summary of EEG studies of FGR/SGA neonates during infancy/childhood.

Study	EEG channel	Popn (N)	Mean GA	PMA of EEG	SWC	Spectral power	Mean EEG amplitude
Cohen et al., 2018	C3–M2, O1–M2 or C4–M1, O2–M1	p-FGR (13)	32^3/7^ (24^6/7^–35^5/7^)	First month; sixth month		↓Relative delta power** was lower in QS at 1 m ↑Relative theta**, alpha** and beta** power were higher in QS at 1 m	
		p-AGA (17)	32 (27^3/7^–35^5/7^)				
Yiallourou et al., 2018	C4–M1, O2–M1, F4–M1	p-FGR (17)	30 ± 1	5–12 years	↑Sleep duration Total—509 mins* ↑NREM—335 mins* ↑N2–198 mins* ↓N3–101 mins* Sleep Proportion ↑N2–50%* ↓N3–25%*	↑Total*, delta*, theta*, and alpha* power were higher	
		p-AGA (15)	29 ± 1		Sleep duration Total—497 min NREM—295 mins N2–154 min N3–112 min Sleep proportion N2–42% N3–32%		
Ozdemir et al., 2009	10–20 system	t-SGA (40)	39.4 ± 0.8	first month; third month		↑Relative delta power 88*/89%*** 1 m/3 m at CzC4 in QS	↓30***/38*** 1 m/3 m
		t-AGA (20)	39.7 ± 0.7			Relative delta power 85/83% 1 m/3 m at CzC4 in QS	66/74 1 m/3 m

**↑** indicate an increase in the SGA/FGR group, **↓** indicate a decrease in the FGR/SGA group.

*GA* gestational age, *PMA* postmenstrual age, *SWC* sleep–wake cycle, *NREM* non-rapid eye movement, *N3* deep sleep, prefix *t* term, prefix *p* preterm, *QS* quiet sleep.

*p < 0.05; **p < 0.01; ***p < 0.001.

Ozdemir et al. found decreases in relative delta power in quiet sleep in SGA neonates within the first week of life compared to AGA controls evolved to increases in relative delta power at 1 and 3 months of age.^44^ This was due to a combination of increases in relative delta power with age in SGA infants and decreases in relative delta power with age in AGA infants.

Cohen et al. performed a longitudinal study of preterm FGR and AGA infants at 1 and 6 months of age with additional term AGA infants as controls.^46^ While measures of growth such as bodyweight had normalised by 1 month of age, there were still notable differences in the EEG spectrum of preterm FGR infants. In quiet sleep, FGR infants had lower spectral edge frequency due to decreased delta power and subsequent increases in theta, alpha, and beta power compared with preterm AGA controls. These differences were not apparent at 6 months of age. No differences were found in active sleep between preterm FGR, preterm AGA and term AGA controls. A key difference between preterm FGR and AGA groups in this cohort was the proportion of neurological injury such as intraventricular haemorrhage (IVH) and periventricular leukomalacia (PVL); 35% of the preterm-AGA group had neurological injuries during their stay in the neonatal intensive care unit compared to no recorded injuries in the FGR group. This imbalance in neurological injuries between FGR and AGA groups is not typical,^47^ but possible when collecting small samples. The unusual distribution of neurological injury in these cohorts should be considered when interpreting EEG findings and maybe why no differences were observed at 6 months of age between cohorts, in contrast to the two studies demonstrating long-term EEG alterations.^44,48^

Yiallourou et al. showed changes in the EEG persist into later childhood. They report several differences in the microarchitecture of sleep (relative spectral band-power within sleep states) between preterm FGR, preterm AGA, and term AGA infants at 5 and 12 years of age.^48^ Interestingly, the sleep microarchitecture of children born prematurely and FGR was more closely related to children born at term and AGA than children born preterm and AGA. This similarity between preterm FGR and term AGA groups was also seen when assessing the macro-architecture of sleep (the relative proportion of sleep states). Yiallourou et al. found no associations between birth weight, head circumference percentiles, or head circumference to weight ratio with any of the sleep microarchitecture measures.^48^

### EEG assessments of interventions on FGR/SGA neonates

Although there are holistic approaches aimed at minimising exposure to modifiable risk factors that are associated with FGR/SGA such as smoking and recreational drug use,^49^ many other risk factors of FGR/SGA such as maternal age at pregnancy, fertility treatments, and stress are not as easily addressed.^50^ Pre-partum clinical trials aimed at improving the growth of fetuses identified as being at risk of FGR/SGA include in utero administration of sildenafil, melatonin, and vascular endothelial growth factor gene therapy.^51–53^ However, limited benefit has been shown by interventions to treat FGR in utero^5^ as have other approaches, such as optimising the timing of delivery.^54^ Difficulties in trialling in utero interventions are exacerbated by difficulties in accurately diagnosing FGR; approximately 40% of FGR/SGA neonates are not detected until birth.^17^ This implies that post-partum interventional studies may better stratify FGR/SGA and AGA neonates. These trials are less common, with the NIDCAP (Newborn Individualized Developmental Care and Assessment Program) trial the most comprehensive in terms of follow-up using EEG analysis.^55^ NIDCAP is a combination of various therapies that aim to “maintain an intimate connection between parents and preterm infants by embedding the infant in the natural parent niche, avoiding over-stimulation, stress, pain, and isolation while supporting self-regulation, competence, and goal orientation”.^56^ The authors analyse the EEG to examine the connectivity between brain regions corresponding to EEG electrode positioning. This analysis breaks EEG activity into a sequence of 40 spatio-frequency patterns that explain 65% of the variance of quiet sleep in a previously collected EEG dataset of 312 neurologically normal infants at 42 weeks PMA.^57^ They found that FGR infants treated with NIDCAP had underlying patterning of the EEG more similar to normal healthy term infants, at 9 months of age, than FGR controls. In particular, they noted reduced connectivity and a higher ratio of long- to short-range connections in FGR infants treated with NIDCAP.^55,58^ Differences in EEG connectivity between treated and untreated infants were still present in the eyes closed, alert state at 9 years of age.^59^

### Neurodevelopmental outcomes

FGR and SGA neonates present with multiple neurodevelopmental deficits into childhood.^3,14,60^ Three studies examined the correlation between EEG/aEEG and neurodevelopmental outcomes in FGR/SGA neonates.

Schwindt et al. performed aEEG at <14 days for 3 hours in a preterm SGA cohort.^33^ Neurodevelopment was assessed with Bayley Scales of Infant Development Second Edition (Bayley II) and a standardised neurologic examination including gross motor function classification system (GMFCS) at 2 years of age. Preterm SGA infants had poorer neurodevelopmental outcomes at 2 years and were more likely to develop multiple complications of prematurity, such as IVH (37.5%), PVL (20.5%), chronic lung disease (30.8%), necrotising enterocolitis (NEC; 30.8%), CP (25.7%), epilepsy (10.3%), and death (21.3%). A correlation between aEEG and neurodevelopmental outcome was observed in both SGA and AGA groups; aEEG correlated with neurodevelopmental outcome more closely in AGA than SGA. The authors speculate that this reduction in correlation could be due to the high morbidity rate and small sample size in the SGA cohort. They suggested earlier, longer recording could improve the prognostic value of this method.

Castro Conde et al. performed continuous EEG at 48–72 hours after birth in a term SGA cohort.^43^ Neurodevelopment was assessed with Bayley Scales of Infant Development Third Edition (Bayley III) at 2 years of age. Lower neurodevelopmental scores were evident in the SGA children compared with the AGA cohort. High rates of particular EEG patterns were associated with lower scores in one or more neurodevelopmental scores. A negative correlation between language scores and relative alpha power and a positive correlation between delta, alpha ratio with language, and motor scores were reported.

Yerushalmy-Feler et al. performed aEEG within 48 h of birth for 3 h in a preterm FGR cohort.^41^ Neurodevelopment was assessed with the Lacey Assessment of the Preterm Infant (LAPI) before discharge, after reaching the 35^th^ gestational week. LAPI pre-discharge examination demonstrated significant differences in tone and development score (sum of all scores) in the FGR group in comparison to AGA but no differences in the motor and neurologic scores. aEEG bandwidth was negatively correlated with the tone, motor, and development scores and relative delta power was negatively correlated with oral motor, tone, and development scores.

Although all three studies demonstrated correlations in certain domains of neurodevelopmental assessments with EEG/aEEG in FGR/SGA cohorts, the predictive values of these outputs regarding developmental deficits were not defined. Heterogeneity in study design also limits the ability to draw conclusions between studies.

### Strengths and limitations

All studies show EEG/aEEG differences between SGA and AGA infants. These differences are present at different stages of development and persist into later childhood. Interestingly, there are changes in EEG in preterm SGA infants without clear signs of early neurological injury.^9,33^

In general, according to the STROBE checklist, these studies were of high quality (Table S1). Studies analysed were prospectively designed, used sufficient sample sizes, included control AGA groups (group-level matched), and several efforts to control for confounders (reducing study bias). Nevertheless, there are inherent difficulties associated with studying critically ill infants in the NICU where interventions related to infant care increase the variability within study cohorts.

The key sources of heterogeneity between these studies were GA and postmenstrual age (PMA) at the time of aEEG/EEG recording. GA ranged from extremely preterm to term, PMA ranged from <72 h to 4 weeks after birth, with later recordings up to 9 years of age. There are rapid changes in aEEG/EEG with GA, ex utero vs in utero exposure, and post-natal adaption.^40,61,62^ Prematurity is independently associated with neurological maladaptation and subsequent changes in the EEG.^63,64^ Premature infants are at a high risk of several, acute and chronic injuries from IVH to an encephalopathy of prematurity.^65,66^ This increased risk also manifests in FGR infants with preterm FGR infants at higher risk of abnormal neurodevelopmental outcome than term FGR infants.^14^ No studies compared the difference between AGA and SGA/FGR on cohorts of both term and preterm infants.

An additional source of heterogeneity between SGA and AGA infants that may confound the interpretation of the aEEG is general infant health; SGA infants tended to have poorer health outcome.^33,41,43,46^ Only two studies excluded infants with abnormal neurodevelopmental outcome^43,44^ nevertheless, they showed differences in the EEG between SGA and AGA infants. Schwindt et al. performed additional analysis on subgroups of SGA and AGA infants without sedatives or the manifestation of cerebral lesions and showed similar differences between SGA and AGA groups.^33^ They also showed early preterm SGA infants had higher rates of morbidity and mortality compared to AGA infants. In aEEG analyses, there were differences in definitions and granularity of aEEG grades, with the predominant definitions based on the influential work of Hellstrom-Westas et al.^67^ The larger array of summary measures that can be estimated from multi-channel EEG results in increased heterogeneity. While most studies used spectral power and measures of sleep architecture, each study examined unique measures such as burst frequency, inter-burst interval, asymmetry, asynchrony, and amplitude. Another key difference between SGA and AGA infants that may affect the EEG is the smaller head circumference in SGA infants—EEG amplitude, and potentially spectral power, are altered by the proximity of electrodes used to form the bipolar montage studied.^68^ There were also technical differences between studies in the electrode locations used for spectral analysis and the definition of frequency bands.

These sources of heterogeneity do not explicitly affect study conclusions, but rather complicate the interpretation of the literature, where superficially contradictory findings across several studies can be explained by cohort differences such as the GA of the infants, PMA of the EEG recording, and differences in EEG analyses. This difficulty limits meta-interpretations of the data across studies.

Heterogeneity is also apparent in the definition of FGR/SGA. Although SGA fetuses are physiologically small, they are at lower risk for adverse perinatal outcomes compared to FGR fetuses. Most of the studies use a definition of SGA as a body weight <10th percentile of local norms with others using additional conditions such as middle cerebral artery Doppler assessment and biometric measurements of the foetal ultrasound. No study conforms to recent attempts to standardise the definition of FGR,^2^ although several use many of the criteria. Three of the studies in this review report their population are FGR,^41,46,48^ with the others reporting on SGA.^9,33,40,43,44^ However, some of the SGA cohorts may indeed be FGR according to the definition of the population.^43^ A further source of heterogeneity in the FGR cohort is the FGR phenotype. FGR can generally be classified as early- or late-onset with these two different forms resulting in differences in neurodevelopmental outcomes.^69^ Only one of the studies classified their cohort as late-onset FGR.^41^ Cohen et al. report slowing of foetal growth velocity in their cohort which may implicate late-onset FGR.^70^ Finally, there was obvious heterogeneity in the tools used to define neurodevelopment/disability. The use of age-appropriate standardised tests of neurodevelopmental outcomes limited comparisons between studies.

All studies used visual interpretation of the EEG with a proportion complemented with quantitative EEG analysis (eg spectral power). The visual interpretation of the EEG is well studied but subject to inter-rater variability. Quantitative EEG is objective, can be consistently applied across EEG studies, and can represent EEG characteristics that may not be possible with visual interpretation.^71,72^ Quantitative EEG analyses are also well-suited to standardisation improving study heterogeneity. Furthermore, improving the accessibility of the EEG recordings would alleviate study heterogeneity. This could be achieved by using centralised repositories where researchers across different centres can upload aEEG/EEG recordings along with clinical data (although issues around patient privacy complicate this process).

### Summary

There is growing evidence from clinical and epidemiological studies that neurodevelopmental disabilities such as CP, learning, attention, and behavioural disorders result from a prenatal chronic insult such as FGR.^3,11,73^ EEG/aEEG is an effective way of assessing brain development in infants and children and is different between FGR/SGA and AGA infants. Changes in EEG were generally consistent across studies with FGR/SGA resulting in measurable effects on the EEG that present immediately after birth and persist into childhood. Early manifestations of FGR/SGA in the EEG include changes in spectral power, symmetry/synchrony, sleep–wake cycling, and the continuity of EEG amplitude. Later manifestations of FGR/SGA in the EEG include changes in spectral power, sleep architecture, and EEG amplitude.

Future research will help to determine if FGR results in a relatively normal brain with delayed maturation, a fundamentally different brain, or merely a brain that is more susceptible to architecture-altering insults. This is challenging, as the links between brain development, disruption, and cortical activity (as measured by the EEG) are predominantly circumstantial.^74^ The diagnostic and prognostic utility of EEG for FGR/SGA infants also requires further elucidation to determine which combination of EEG/aEEG characteristics and timeframes of measurement provide the best discrimination between FGR/SGA and AGA infants. If this separation is sufficiently large, EEG criteria may be useful to complement definitions of FGR/SGA, and normalisation of the EEG may be considered as an outcome measure for intervention effect. Of particular interest, is the use of EEG in FGR/SGA infants for the early prediction of adverse neurodevelopment. Incorporating EEG biomarkers that are associated with SGA/FGR with other measures of abnormal EEG^75^ also offer the possibility of accurate prognostication.^72–76^

Automated methods of analysis have been applied to neonatal EEG; automatically extracting information from the preterm EEG.^27,77^ No automated methods have been used in the FGR/SGA neonate so far. However, even though automated methods are appealing caution is required when using these modern techniques, as the recordings can be influenced by several factors which must be considered for accurate interpretation, including the behavioural state of the infant and artefact.^78^ Nonetheless, the EEG holds promise as a tool to achieve early diagnosis and prognosis in FGR/SGA neonates.

## Supplementary information

Supplementary Table
