# Supplementary material for: Electroencephalographic studies in growth-restricted and small-for-gestational-age neonates
Source: Pediatr Res. 2022 Feb 23;92(6):1527–34. doi: 10.1038/s41390-022-01992-2 (PMC9771813; doi:10.1038/s41390-022-01992-2)
Supplement: Supplementary file 1 — Supplementary Table [file 41390_2022_1992_MOESM1_ESM.docx]

| **STROBE CHECKLIST** | Schwindt 2015 | Benavente-Fernandez 2017 | Griesmaier 2015 | Yerushalmy-Feler 2014 | Castro Conde 2020 | Ozdemir 2009 | Cohen 2018 | Yiallourou 2018 |
| --- | --- | --- | --- | --- | --- | --- | --- | --- |
| Title and abstract | Y | Y | Y | Y | Y | Y | Y | Y |
| Background/rationale | Y | Y | Y | Y | Y | Y | Y | Y |
| Objectives | Y | Y | Y | Y | Y | Y | Y | Y |
| Study design | Y | Y | Y | Y | Y | Y | Y | Y |
| Setting | Y | Y | Y | Y | Y | N | Y | Y |
| **Participants (methods)** |  |  |  |  |  |  |  |  |
| a) Eligibility/Follow Up | Y | Y | N | Y | Y | Y | Y | Y |
| b) Matched Studies | N/A | N/A | N/A | N/A | N/A | N/A | N/A | N/A |
| Variables | Y | Y | Y | Y | Y | Y | Y | Y |
| Data sources | Y | US | Y | Y | Y | Y | Y | Y |
| Bias | Y | US | N | Y | Y | Y | Y | Y |
| Study size | N | Y | N | Y | Y | Y | Y | Y |
| Quantitative variables | Y | Y | Y | Y | Y | Y | Y | Y |
| **Statistical methods** |  |  |  |  |  |  |  |  |
| a) Describe Methods | Y | Y | Y | Y | Y | Y | Y | Y |
| b) Subgroups/Interactions | Y | Y | N/A | Y | Y | N | N | Y |
| c) Missing Data | N/A | N | N | N/A | N/A | N | N | N/A |
| d) Lost-to follow up | N | N/A | N/A | N | Y | N/A | N/A | N/A |
| e) Sensitivity Analyses | N | N | N | N | N | N | N | N |
| **Participants (results)** |  |  |  |  |  |  |  |  |
| a) Numbers | N | Y | Y | N | Y | Y | Y | Y |
| b) Reasons for non-participation | N | N | Y | N | Y | Y | N | Y |
| c) Flow diagram | N | N | N | N | Y | N | N | N |
| **Descriptive data** |  |  |  |  |  |  |  |  |
| a) Demographics | Y | Y | Y | Y | Y | Y | Y | Y |
| b) Missing data | N/A | N | N | N/A | N/A | N | N | N |
| c) follow-up time | Y | Y | N/A | Y | Y | Y | N/A | N |
| Outcome data | Y | Y | N | Y | Y | Y | N | N |
| **Main results** |  |  |  |  |  |  |  |  |
| a) Unadjusted estimates / confounder adjusted | N | N/A | N/A | N | N | N/A | N | N |
| b) Category boundaries | Y | N | Y | N/A | Y | N/A | N/A | N/A |
| c) Absolute risk instead of RR | N/A | N/A | N/A | N/A | N/A | N/A | N/A | N/A |
| Other analysis | Y | Y | Y | Y | Y | Y | Y | Y |
| Key results | Y | Y | Y | N | Y | Y | Y | Y |
| Limitations | Y | Y | Y | Y | Y | Y | Y | Y |
| Interpretation | Y | Y | Y | Y | Y | Y | Y | Y |
| Generalisability | Y | Y | Y | Y | Y | Y | Y | Y |
| Funding | Y | Y | Y | Y | Y | N | Y | Y |
